# Designer natriuretic peptides for heart failure: advanced in clinical development

**DOI:** 10.1186/1471-2210-11-S1-O1

**Published:** 2011-08-01

**Authors:** John Burnett

**Affiliations:** 1 Mayo Clinic, Mayo Heart and Lung Research Center, Rochester, USA

## Background

The increasing burden of heart failure (HF) is a major unmet need for new therapeutics. No new drug has been approved for HF for over a decade. The natriuretic peptides as endogenous activators of particulate guanylyl cyclase (GC) receptors represent an unparalleled opportunity for novel therapeutic agents for HF. Specifically, the GC-A receptor activated by ANP and BNP and GC-B activation by CNP mediate beneficial pleiotropic actions, which include natriuresis, vasodilatation, suppression of the renin-angiotensin-aldosterone system, inhibition of cardiomyocyte hypertrophy, retardation of fibrosis and improvement in ventricular function and structure.

## Results

CD-NP (Cenderitide) represents the first in class designer natriuretic peptide which co-activates both GC-A and GC-B. Through rational drug design to co-activate GC-A and GC-B together with increased resistance to peptidase degradation, CD-NP possesses actions which make CD-NP an innovative protein therapeutic to target the heart, kidney, vasculature and endocrine system in HF to reduce rehospitalization following acute decompensated HF.

## Conclusion

Here we review the biology, preclinical development and recent human studies advancing CD-NP as a chronic peptide therapeutic designer peptide to reduce the risk of rehospitalization in human chronic HF.

